# Enhanced Recovery of Oil Mixtures from Calcite Nanopores Facilitated by CO_2_ Injection

**DOI:** 10.1021/acs.energyfuels.3c05235

**Published:** 2024-03-08

**Authors:** Hongwei Zhang, Shihao Wang, Xin Wang, Rui Qiao

**Affiliations:** † Department of Mechanical Engineering, Virginia Tech, Blacksburg, Virginia 24061, United States; ‡ Chevron Technical Center, Chevron, Houston, Texas 77002, United States

## Abstract

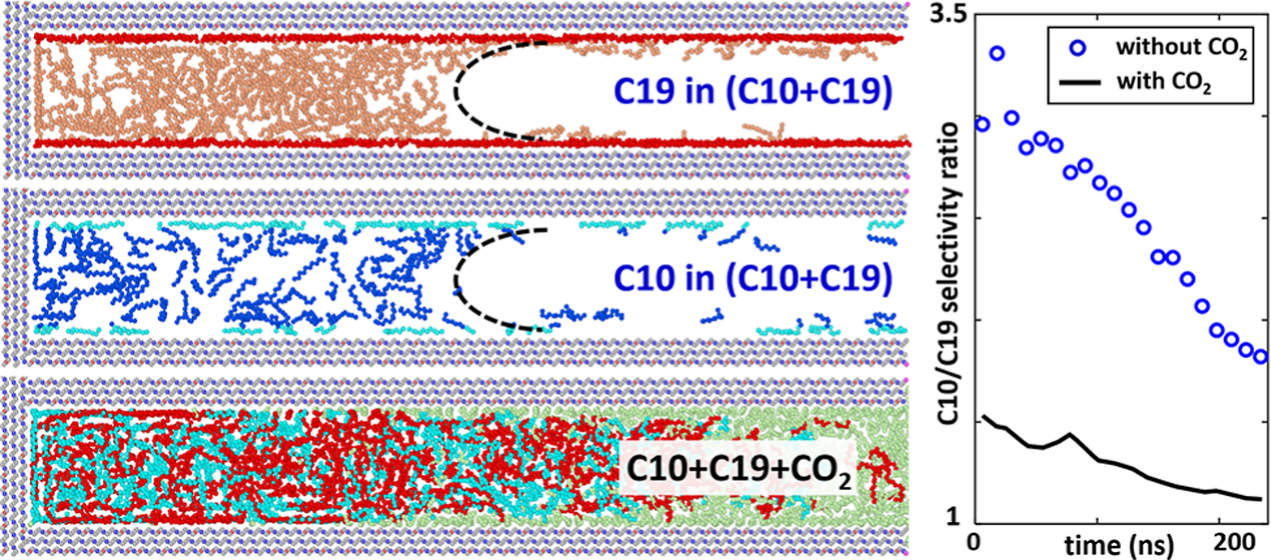

Slow production, preferential recovery of light hydrocarbons, and low recovery factors are common challenges in oil production from unconventional reservoirs dominated by nanopores. Gas injection-based techniques such as CO_2_ Huff-n-Puff have shown promise in addressing these challenges. However, a limited understanding of the recovery of oil mixtures on the nanopore scale hinders their effective optimization. Here, we use molecular dynamics simulations to study the recovery of an oil mixture (C10 + C19) from a single 4 nm-wide calcite dead-end pore, both with and without CO_2_ injection. Without CO_2_ injection, oil recovery is much faster than expected from oil vaporization and features an undesired selectivity, i.e., the preferential recovery of lighter C10. With CO_2_ injection, oil recovery is accelerated and its selectivity toward C10 is greatly mitigated. These recovery behaviors are understood by analyzing the spatiotemporal evolution of C10, C19, and CO_2_ distributions in the calcite pore. In particular, we show that interfacial phenomena (e.g., the strong adsorption of oil and CO_2_ on pore walls, their competition, and their modulation of transport behavior) and bulk phenomena (e.g., solubilization of oil by CO_2_ in the middle portion of the pore) play crucial roles in determining the oil recovery rate and selectivity.

## 1 Introduction

Shale and tight oil reservoirs with massive hydrocarbon storage have emerged as a frontier in the global energy landscape, and their exploration and exploitation have expanded significantly in recent decades.^1^ However, production from these unconventional reservoirs still faces many challenges. These reservoirs have a low porosity, often dominated by nanoscale pores, resulting in extremely low permeability. Such low permeability causes a rapid decline in oil production during the primary depletion, and the oil recovery factor is below, if not far below, 7%.^2^ To reduce the revenue loss associated with low oil recovery factors, many enhanced oil recovery (EOR) methods have been developed to increase oil recovery in unconventional oil reservoirs.^3,4^

Among the proposed methods, the EOR by gas injection has attracted significant attention. Typical choices of injected gases include light hydrocarbons (e.g., CH_4_ and/or C_2_H_6_), CO_2_, and N_2_.^4^ The gas injection-based EOR in unconventional reservoirs is often performed through the Huff-n-Puff process.^4−7^ In the Huff cycle, gas is injected into the reservoir through the production well until the downhole pressure reaches a certain threshold. Then, the production well is shut during the soaking cycle. The injected gas permeates fractures and nanopores in the reservoir. As the permeation proceeds, the gas may cause the oil to swell and lower its viscosity, thus mobilizing it from the rock matrix to the more conductive fracture network. After the soaking step, the production well is reopened, and oil and some injected gas are produced in the Puff cycle.^7^ Gas-based Huff-n-Puff has seen successful applications in many fields, although failed cases have also been reported.^3,6,8−10^ To exploit the full potential of Huff-n-Puff, a comprehensive understanding of its underlying physics is needed.

The physical processes involved in gas-based Huff-n-Puff depend on the nature of the crude oil in unconventional reservoirs. Oils in these reservoirs contain hydrocarbons with a broad spectrum of molecular weights, e.g., those from the Bakken play contain a significant mole fraction of C1 and C5 to C36.^11^ Because these hydrocarbons interact differently with the injected gases, they have different solubilities in them, and their relative mole fraction governs oil–gas mixture properties such as minimum miscibility pressure, viscosity, and diffusivity.^7,12−15^ Therefore, the recovery of hydrocarbons of various molecular weights from reservoirs and its enhancement by gas injection are expected to differ. Understanding the selective recovery of hydrocarbons is crucial to optimizing Huff-n-Puff operations in practice.

Experimental studies aiming at understanding selective oil recovery during the Huff-n-Puff process have emerged in recent years. In core-scale tests, Hawthorne et al. exposed rock samples to CO_2_ and revealed that lighter hydrocarbons (C7 to C14) are recovered faster than the heavier hydrocarbons (>C20) from all samples.^16,17^ The results suggest that, during CO_2_-EOR, crude oil does not move as a homogeneous phase; instead, substantial deposition of heavy hydrocarbons on pore walls in the sample can occur, and a strong selectivity of hydrocarbon recovery is expected.^16,17^ Through a series of experiments, they concluded that, in addition to higher diffusivity, the greater solubility of lighter hydrocarbon contributes to the selective recovery of different oil components.^18^

While the above studies revealed the importance of the *bulk behavior* of oil–gas mixtures in the selective recovery of hydrocarbons from unconventional reservoirs, other studies highlighted the importance of *interfacial behavior*. In core-scale studies, Zhu et al. evaluated the recovery of a C10–C17 binary mixture through Niobrara shale samples and found that the recovery of C17 is lower than that of C10.^19^ Such selectivity is significantly mitigated when CO_2_ is introduced. These phenomena are understood by noting that due to the prevalence of nanopores in shales, a significant fraction of oil can interact strongly with pore walls, and such fluid–wall interactions modulate oil recovery. Specifically, in the absence of CO_2_, the heavier C17 molecules adsorb more strongly on the pore walls, which retard their recovery and enable preferential C10 recovery. When CO_2_ is introduced, as showed by equilibrium molecular dynamics (MD) simulations,^20^ C17 molecules are displaced from the pore walls by CO_2_ molecules, and thus the preferential recovery of C10 is suppressed.

Besides experimental studies, theoretical and computational studies can provide insights into the selective recovery of hydrocarbons from unconventional reservoirs, including when the recovery is aided by gas injection. In this regard, MD simulations are instrumental in probing how interfacial phenomena affect such a recovery. MD simulations have been used to study the selective adsorption of hydrocarbon on pore walls^21−23^ and the alteration of flow behaviors by adsorbed molecules,^24−28^ although most available work focused on gaseous rather than liquid hydrocarbons. For example, the recovery of different hydrocarbons from dead-end pores^29^ and through pore throat^30,31^ has been studied. These studies showed that the selective adsorption of different components on pore walls and the wall-mediated coupling between the transport of different hydrocarbons are key factors governing the selective recovery of shale gas of different molecular weights from shales.^29^ In particular, Ho and Wang concluded that the surface diffusion of adsorbed shale gas molecules is responsible for the selectivity between methane and ethane when a pore is narrower than 1.8 nm.^30^ Guided by MD simulations, Wang et al. developed an analytical transport model for modeling the differential release of a gas mixture from shale reservoirs.^31^ Their model accounts for the selective adsorption of different gas species on pore walls, slippage flow, and surface diffusion on the pore walls. The model predicts a differential release of multicomponent gas mixtures from shale and tight gas reservoirs, and its prediction has been validated against MD simulations of gas release through a single pore throat.^31^

The previous work has advanced the fundamental understanding of the selective recovery of hydrocarbon mixtures from unconventional reservoirs. Nevertheless, there is a lack of systematic study of the selective recovery of oil mixtures aided by gas injection. Indeed, many questions about such recovery remain open. For example, how are heavy and light hydrocarbons recovered? What are the roles of interfacial phenomena (e.g., adsorption and modulation of transport by fluid–wall interactions) in the selective recovery of oils with different molecular weights? Can CO_2_ injection mitigate the preferential recovery of lighter hydrocarbons and what are the underlying mechanisms? Answering these questions will benefit from time-resolved data on the oil composition and distribution across nanopores during oil recovery, especially on the single-nanopore scale. Answering these questions will also help guide the introduction of nanoscale physics into oil recovery models beyond those considering mainly thermodynamic effects (e.g., equation of state models such as the nanoPVT).^32−34^

In this work, we use MD simulations to study the recovery of a C10–C19 binary mixture from a dead-end nanopore by the use of CO_2_ injection. The rest of this paper is organized as follows. Section 2 presents the MD systems and methods. Section 3 presents the recovery behaviors of C10 and C19 and the underlying spatiotemporal evolution of the oil and CO_2_ density inside the nanopore. The selective recoveries of C10 and C19 without and with CO_2_ are compared, and their underlying mechanisms are elucidated. Finally, conclusions are drawn in Section 4.

## 2 Simulation System, Protocol, and Methods

### 2.1 System for Oil Recovery

**Figure 1:**
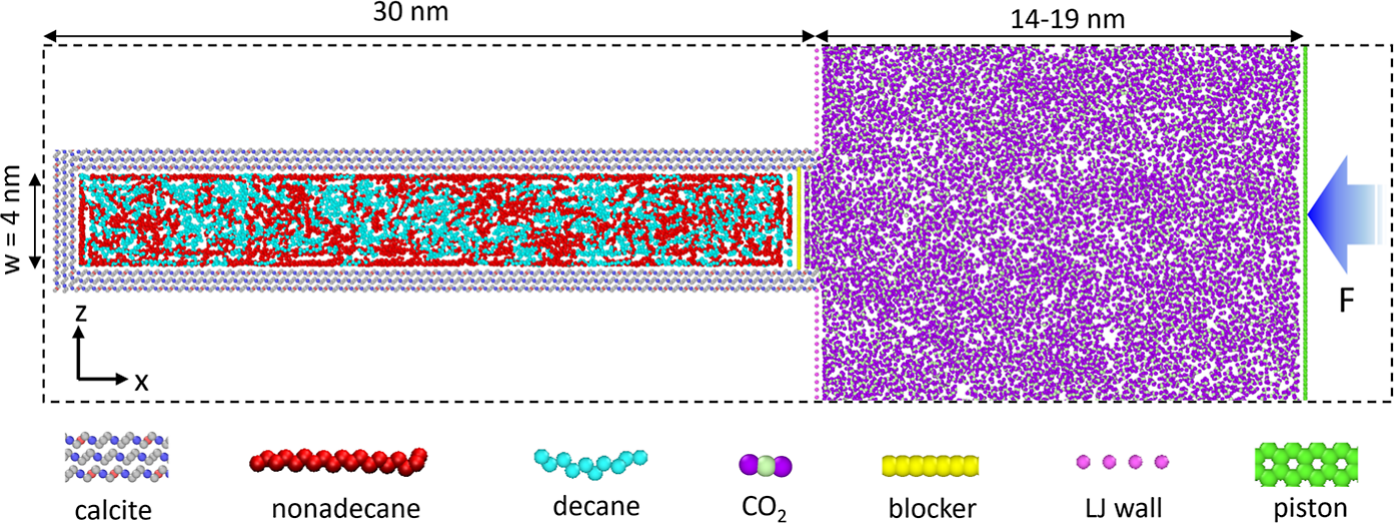
Snapshot of the simulation system for studying the recovery of decane + nonadecane (C10 + C19) mixtures from a single calcite nanopore aided by CO_2_ injection. The system measures 29.46 nm in the *z*-direction, and only part of the gas bath is shown to save space (see a full snapshot of the system in Figure S1 of the Supporting Information). The dashed black lines denote the simulation box.

Our MD system is designed to investigate the recovery of residual oil from a dead-end nanopore aided by gas injection. As shown in Figure 1, the system consists of a slit-shaped calcite nanopore connected to a gas bath, whose pressure is controlled by a piston. The pore width is 4 nm, consistent with the fact that nanopores are ubiquitous in shale oil reservoirs. The pore length is set to 30 nm so that the slender shape of nanopores in real shales is captured reasonably well. Initially, the nanopore is filled with a binary oil mixture, and the bath is filled with gas. Crude oils have complicated compositions. Here, C10 and C19 are selected to represent crude oil’s light and heavy components. The initial C10-to-C19 mol ratio inside the nanopore is set as 1.4:1, close to that found in an unconventional Bakken Formation reservoir in the Williston Basin.^11^ The gas bath is filled with CO_2_ to probe the effect of CO_2_ injection on selective oil recovery from the nanopore. As a reference, we consider the case in which the gas reservoir is empty. A vacuum is placed on the blocker’s right side.

The simulation box measures 100.00, 2.93, and 26.46 nm in the *x*, *y*, and *z*-directions, respectively. The system is periodic in all directions. The *y*-length of the simulation box is chosen to minimize the computational cost and avoid the finite size effect. The recovery of C10 and C19 molecules from a nanopore is mainly controlled by their effective size pertinent to their movement, which can be characterized by using their radius of gyration. From our simulation trajectories, the radii of gyration of C10 and C19 are ∼0.37 and 0.60 nm, respectively. Since these values are much smaller than the *y*-length of the simulation box (2.93 nm), the length of the simulation box in the *y*-direction should be adequate.

### 2.2 Protocol for the Oil Recovery Study

Before conducting the oil extraction simulation, separate simulations are first performed to determine the number of C10 and C19 molecules in the nanopore needed to produce the desired pressure (345 bar) and temperature (373 K). Specifically, we set up a series of simulations in which the number of C10 molecules in the nanopore differed but the mole ratio of C10 and C19 is kept at 1.4. In these simulations, blocker atoms in Figure 1a are placed at the pore entrance, and the pressure on the blocker is measured. The system in which the pressure on the blocker matched the desired pressure (345 bar) is then selected. By trial and error, the numbers of C10 and C19 molecules in the nanopore are determined as 415 and 294, respectively.

Next, the nanopore is packed with C10 and C19 molecules determined above. Following this, the CO_2_ molecules are packed into the gas bath, and a constant force is applied to the piston to maintain a pressure of 345 bar (in the gas-free reservoir case, the piston is fixed in space). The system is equilibrated at 373 K for 5 ns.

After the above preparations, the blocker atoms in Figure 1a are removed at *t* = 0, and a 240 ns production run is performed while the fluid temperature is maintained at 373 K. By the end of the production run, less than 2% of the initial C10 molecules remain inside the pore. Initially, the piston is ∼19 nm from the pore entrance. As CO_2_ enters the nanopore, the piston moves gradually toward the pore entrance and eventually reaches about 15 nm from the pore entrance at the end of the simulation. During the entire simulation, the piston is at least 15 nm away from the pore and the vertical walls. This distance is far larger than the diameter (∼0.4 nm) and the mean free path length of CO_2_ molecules inside the gas bath (<1 nm). Therefore, a bulk behavior is always maintained inside the gas bath.

During each production run, any oil molecule reaching the gas bath is deleted. Furthermore, the pressure in the gas bath is maintained at 345 bar by applying a constant force on the piston. With these operations, the chemical potential of the gas inside the bath is maintained during the oil recovery process.

### 2.3 Molecular Models

The 0.75 nm thick calcite pore wall is cut in the [1014] direction and fixed during the simulations. The partial charges and Lennard-Jones (LJ) parameters of the calcite wall are from the refitted Doves’ potential.^35^ The NERD force field models the alkane molecules in this work with the CH_3_ and CH_2_ motifs treated as united atoms.^36^ The force field for CO_2_ molecules was developed by Zhu et al.^37^ with parameters optimized by Wang et al.^38^ These force fields are chosen based on their capabilities to describe the mixing and phase equilibrium behaviors of the oil–CO_2_ mixture accurately.^39^ The piston and blocker atoms are modeled as LJ atoms arranged in a square lattice to prevent the oil and gas molecules from crossing them. The force field parameters used in this work can be found in Table S1 in the Supporting Information. The Lorentz–Berthelot combination rule is applied to the interaction between other dissimilar atom pairs.

### 2.4 Simulation Methods

All MD simulations in this study are performed using the LAMMPS code in the *NVT* ensemble (note that, under the action of the piston, the volume of the gas bath changes during the production run).^40^ The temperature of the fluids is maintained at 373 K with a Nose–Hoover thermostat. The velocities of fluids in all three directions were thermostated because the collective velocity of the fluids in all directions (especially the *x*-direction) is small. The nonelectrostatic interactions are computed with a cutoff length of 1.2 nm. Electrostatic interactions are handled using the PPPM, with a real-space cutoff length of 1.2 nm and an accuracy of 10^–4^. All simulations are conducted with a time step of 1 fs.

## 3 Results and Discussion

**Figure 2:**
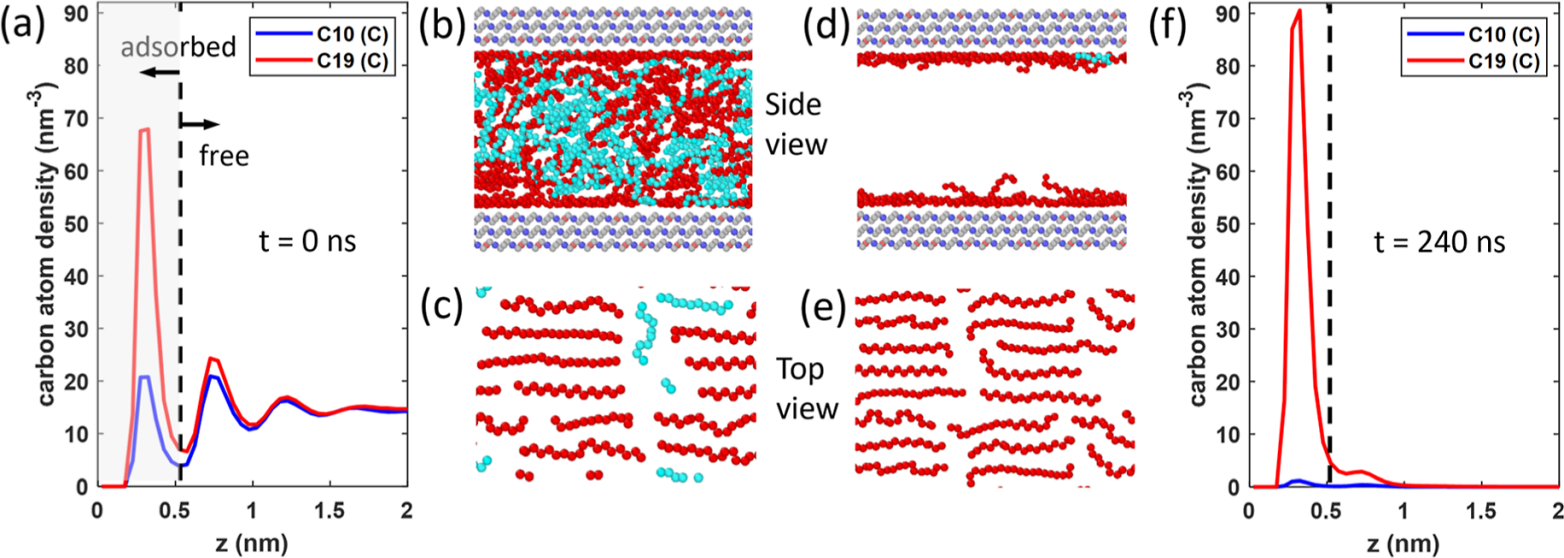
Oil distributions in a calcite slit nanopore at the beginning and end of the oil recovery without gas injection. (a,f) Density profiles of C10 and C19’s carbon atoms across the pore at the simulation’s beginning (a) and end (f). The density profiles are averaged along the entire nanopore and shown only in the lower half of the pore due to symmetry. (b–e) Snapshots of the representative side-view of the oil mixture inside the pore and top-view of the contact-adsorbed oil molecules at the beginning (b,c) and end (d,e) of the oil recovery simulation. In (a,f), *z* = 0 corresponds to the position of the uppermost oxygen atoms of the lower calcite wall.

Before examining the oil recovery from nanopores, let us first inspect the storage of the C10–C19 mixture in the pore at *t* = 0. Figure 2a shows the density profiles of C10 and C19 averaged along the pore length as a function of the distance from the lower pore wall (data in the upper half of the pore are not shown due to symmetry). Three layers of C10 and C19 molecules are identified near the pore wall. The first layers (<0.53 nm) correspond to contact-adsorbed C10 and C19 molecules and are the most distinct. As shown in the side and top views of the system (see Figure 2b,c), the adsorbed C10 and C19 molecules are highly stretched and prefer to adopt a coplanar structure on the wall. C19 adsorption is much stronger than C10 adsorption because, though the densities of the carbon atoms of C10 and C19 in the pore’s bulk portion are comparable, the first C19 peak is more than 3 times higher than the first C10 peak. The stronger adsorption of C19 is attributed to its stronger van der Waals interactions with pore walls. Despite the stronger affinity of C19 to the pore wall, a modest amount of C10 is adsorbed on the pore wall. This is because, from an entropic perspective, the smaller C10 molecules do have an advantage for adsorption: it is easier for them to occupy the interstitial space on the pore walls formed by the adsorbed C19 molecules (Figure 2c).

Given the distinct adsorption of C10 and C19 on pore walls, following convention,^13,24,30,32,34,41^ we divide each component in the pore into two populations: the “adsorbed” oil and the “free” oil (i.e., those on the left and right sides of the dashed line in Figure 2a). As we will see later, from a transport perspective, the oil molecules in the second adsorption layer (*z* = 0.53–0.97 nm) are much more mobile than those in the first adsorption layer and are relatively close to those of bulk oil. This is especially notable in the case without gas injection, where the molecules in the second layer mainly contribute to oil recovery. Therefore, we define oil molecules beyond the first adsorption layer as “free” molecules. The adsorbed population accounts for 15.2 and 32.6% of all C10 and C19 inside the pore, respectively. As we shall see, the different fractions of the adsorbed populations of C10 and C19 contribute significantly to their selective recovery from the pore.

### 3.1 Oil Recovery without Gas Injection

In this reference case, there is no gas in the bath. Upon removal of the blocker atoms at the pore entrance, driven by the pressure difference between the pore oil (345 bar) and the gas bath, the liquid oil mixture in the pore flows toward the bath and any C10/C19 molecules that reached into the gas bath are deleted and considered recovered. However, given oil’s low compressibility,^42^ the pore pressure quickly relaxes, and the collective oil flow toward the gas bath diminishes. The recovery of oil from the pore is thus similar to the loss of oil trapped inside the shale core samples exposed to a low-pressure environment. The present case is also relevant to the situation where condensates are trapped in unconventional reservoirs during the final stage of the primary recovery,^41^ when the pressure difference between the pore and fracture can no longer drive oil recovery.

The classical view of this case is that oil is recovered via vaporization. Such a recovery is expected to be slow due to the low vapor pressure of the oil molecules considered here. Kinetic theories predict that the initial evaporation mass flux of oil species *i* is , where *T* is the temperature. *m*_*i*_, *R*, and *p*_sat,*i*_ are the molecular mass, gas constant, and saturation pressure of an oil species *i*, respectively.^43^ The vapor pressures of the C10 and C19 mixtures are not readily available. Nevertheless, the order of magnitude of *J*_C10_ and *J*_C19_ can be estimated from the vapor pressure of neat C10 and C19 (9.5 kPa and 19 Pa)^44,45^ as 361 and 0.53 mol/m^2^·s, respectively. If vaporization occurs across the entire opening of the nanopore, the initial recovery rates of C10 and C19 are estimated as 2.55 and 0.0037 molecules per nanosecond, respectively. As evaporation proceeds, the oil meniscus will recede into the pore, and the recovery rate will decrease due to the additional vapor transport resistance from the pore interior to the gas bath.

**Figure 3:**
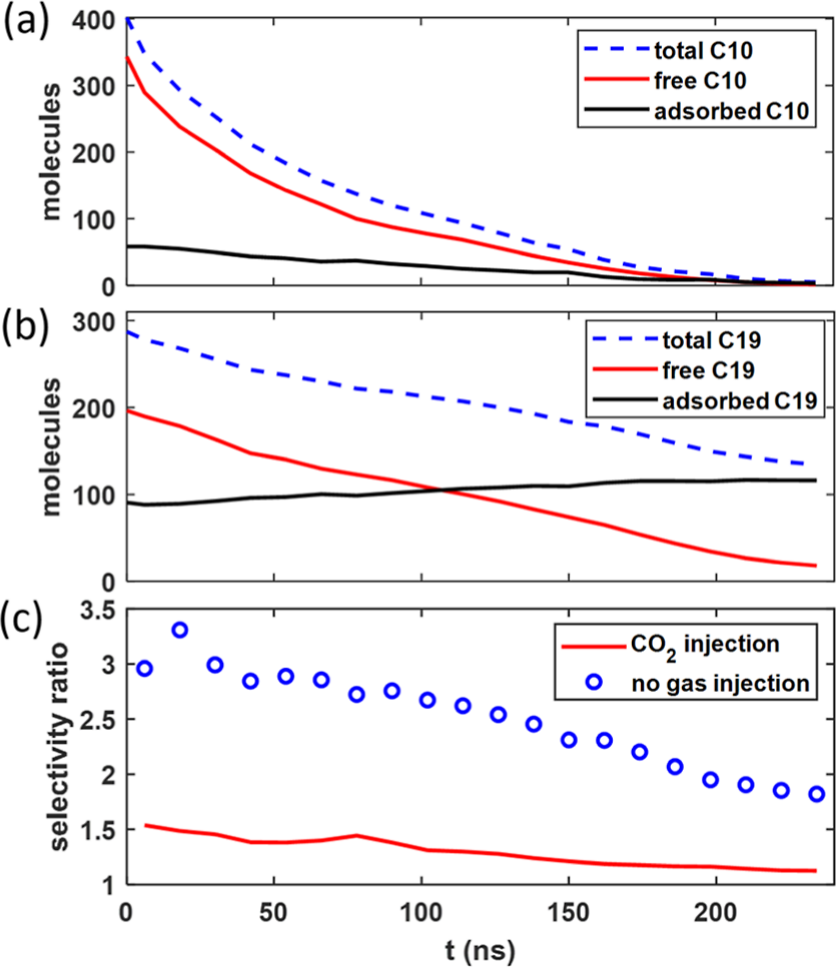
(a,b) Evolution of the number of C10 molecules (a) and C19 molecules (b) and their “free” and “adsorbed” molecules as defined in Figure 2a during oil recovery in the absence of gas injection. (c) Evolution of the selectivity ratio, i.e., the fraction of C10 recovered divided by the fraction of C19 recovered.

Figure 3a,b shows the evolution of the number of C10 and C19 molecules inside the pore observed in our simulations. C10 is recovered at a faster rate than that of C19. At the end of the 240 ns simulations, 98.8% of the C10 molecules are recovered, compared to 55.1% for C19. Equilibrium under the vacuum condition in the gas bath corresponds to the complete removal of C10 and C19 because of the tremendous entropic gain for transferring oil molecules into a vacuum, where the oil concentration is zero (since oil molecules in the bath are removed during our simulations). Because our study focuses on the dynamics of oil recovery, and the oil recovery at *t* > 240 ns is extremely slow, reaching the equilibrium conditions in our simulations is unnecessary.

The initial recovery rates of C10 (C19) molecules are 8.92 ns^–1^ (1.53 nm^–1^), i.e., about 1 (3) orders of magnitude faster than that estimated above. The preferential recovery of C10 over C19 is maintained during the recovery process, indicating a strong selectivity toward lighter oil and the challenge of achieving a high recovery factor for heavy hydrocarbons in unconventional reservoirs. Such undesirable selectivity can be quantified using the selectivity factor, which is defined as the ratio between the fraction of C10 recovered and the fraction of C19 recovered. As shown in Figure 3c, the selectivity factor decreases from an initial value of 3.0 to 1.8 at *t* = 240 ns.

**Figure 4:**
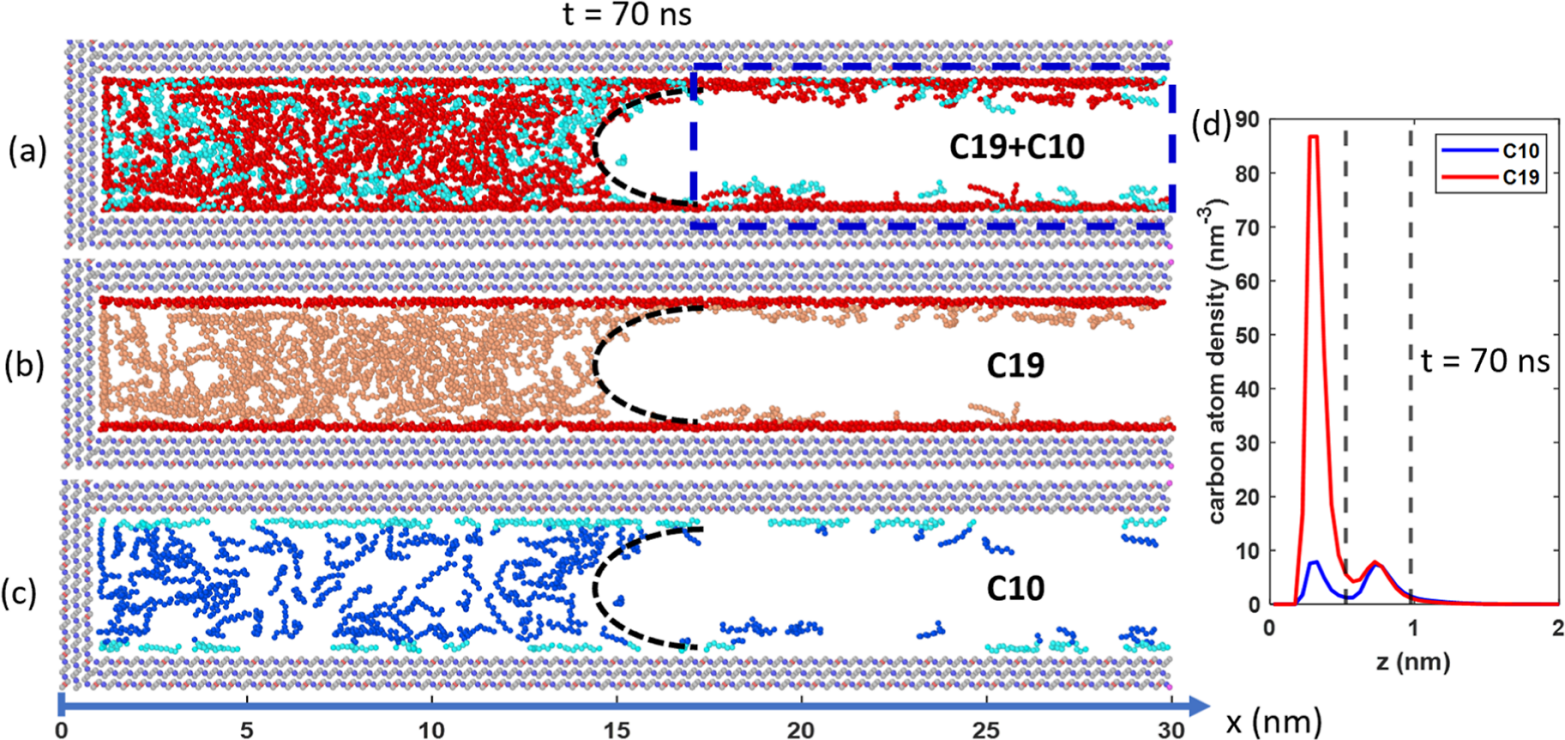
(a–c) Side-view snapshots of the calcite nanopore and oil inside it at *t* = 70 ns of the oil recovery simulation under the no gas injection condition. The black dashed lines are guides for the liquid meniscus. The blue dashed box denotes the space behind the liquid meniscus. (d) Density profiles of C10 and C19 across the calcite pore averaged inside the green box in (a). In (b), red and light red denote the adsorbed and free C19 molecules. In (c), light blue and blue denote the adsorbed and free C10 molecules.

To understand the faster oil recovery compared to that of classical vaporization, we first visualize the recovery process. A representative snapshot of the system, taken at *t* = 70 ns, is shown in Figure 4a. As expected, a liquid meniscus (highlighted by using a dashed line) has emerged and receded into the pore. However, behind the meniscus, molecularly thin films of C10 and C19 appear on the pore walls. Oil molecules move mainly from the contact region between the liquid oil and pore walls into the thin liquid films, diffuse toward the pore opening, and are eventually recovered, which is akin to water imbibition into gas-filled nanopores through surface hydration and diffusion.^46^ Further, the recovery of oil, especially the heavier C19 molecules, occurs mainly through the diffusion of oil molecules in the second adsorption layer due to their far higher mobility.

The transport of oil molecules along the liquid films behind the meniscus toward the gas bath helps explain the faster oil recovery than vaporization. To appreciate this, we compute the density profiles of C10 and C19 behind the meniscus at *t* = 70 ns (i.e., in the region 17 nm < *x* < 30 nm, see the green box in Figure 4a). The two density peaks of C10 in Figure 4d correspond to C10 adsorbed on each wall and C10 adsorbed on top of the contact-adsorbed C10 molecules; similar observations apply to the C19 density profiles. Integration of these density profiles indicates that the amounts of C10 and C19 molecules in these layers, if averaged across the pore width *w*, are 0.170 and 0.413 nm^–3^, respectively (i.e., ρ̅_*i*_ = ∫_0_^*w*^ρ_*c,I*_ (*z*) d*z*/*wn*_*c,i*_, where ρ̅_*c,i*_(*Z*) is the number density of the carbon atoms of oil species *i* shown in Figure 4d. *n*_*c,i*_ = 10 (19) is the number of carbon atoms in each C10 (C19) molecule). Should oil recovery occur via vaporization only, the average density of C10 and C19 across the pore width would be estimated from their saturation vapor pressure to be on the order of 1.85 × 10^–3^ and 3.72 × 10^–6^ nm^–3^, respectively. These data show that pore walls interacting strongly with oil molecules lead to their significant enrichment behind the meniscus moving toward the pore’s interior, thus contributing to faster oil recovery than vaporization alone. The transport of oil molecules adsorbed on pore walls is slower than that of the oil molecules diffusing in the gas phase, but that appears to be a secondary effect here.

The selectivity of C10 over C19 during oil recovery assisted by surface adsorption has several origins. The recovery rate of each species through the liquid films behind the meniscus is governed by its amount inside the liquid films, the chemical potential gradient driving it toward the gas bath, and its mobility. The total amount of C19 inside the liquid films is higher than C10; e.g., at *t* = 70 ns, there are 2.4 times more C19 than C10 molecules. However, our visualization of simulation trajectories reveals that C19 recovery is mainly contributed by molecules in the second adsorption layer (for C10 molecules, which occupy the interstitial space between contact adsorbed C19 and often straddle between the first and second adsorption layers, those in the first adsorption layer are recovered relatively readily, albeit more slowly than those in the second layer). Because C19 is more enriched in the first adsorption layer than in the second adsorption layer (Figure 4b,c, which shows C10 and C19 molecules separately and highlights the adsorbed molecules), the enrichment of C19 in the second adsorption layer is weaker than that of C10. In fact, integration of the second C10 and C19 peaks reveals that the number of C19 molecules in the second adsorption layer is only 0.63 times of C10 molecules there, compared to the C19:C10 ratio of 0.71 in the initial oil mixture. Therefore, the number of C19 molecules in the liquid films participating in oil recovery is smaller than those of C10, thus contributing to the observed selectivity of C10 over C19 during oil recovery. Furthermore, the longer chain of C19 molecules makes them less competitive in recovery than C10 through two other mechanisms: C19 molecules interact more strongly with their surrounding oil molecules and wall atoms, which leads to lower mobility and smaller chemical potential gradient driving their recovery.

Having studied the overall recovery behaviors of C10 and C19, we next examine how the two populations (free vs adsorbed) of oil molecules are recovered from the nanopore. Figure 3a shows that the number of free and adsorbed C10 molecules decreases with time, but the reduction of free C10 dominates C10 recovery given its larger population than adsorbed C10. The recovery of C19 shows a different picture: while the number of free C19 decreases with time and approaches zero, that of adsorbed C19 *increases* with time. The latter is consistent with the observation that the first C19 density peak at the end of the oil recovery simulation is higher than that at *t* = 0 (cf. Figure 2a,f). During oil recovery, adsorbed C19 molecules leave the pores by surface diffusion. However, C19 molecules leaving the liquid meniscus easily become adsorbed on the pore walls before leaving the pore. Further, free C19 molecules also move to vacant spots left by the C10 molecules departing the pore. During the period probed in our simulation, such replenishment of adsorbed C19 occurs faster than the depletion of adsorbed C19 from the pore, thus leading to an increase in the amount of adsorbed C19. At *t* = 240 ns, pore walls are still populated by C19 molecules that conform closely to them (see Figure 2d,e). At a much larger time scale, adsorbed C19 molecules would be recovered, albeit at an even slower rate. In real reservoir operations, even though surface-enhanced recovery may help recover some heavy oil, fully recovering the oil contact adsorbed on the pore walls is likely impractical.

### 3.2 Enhanced Oil Recovery with CO_2_ Injection

**Figure 5:**
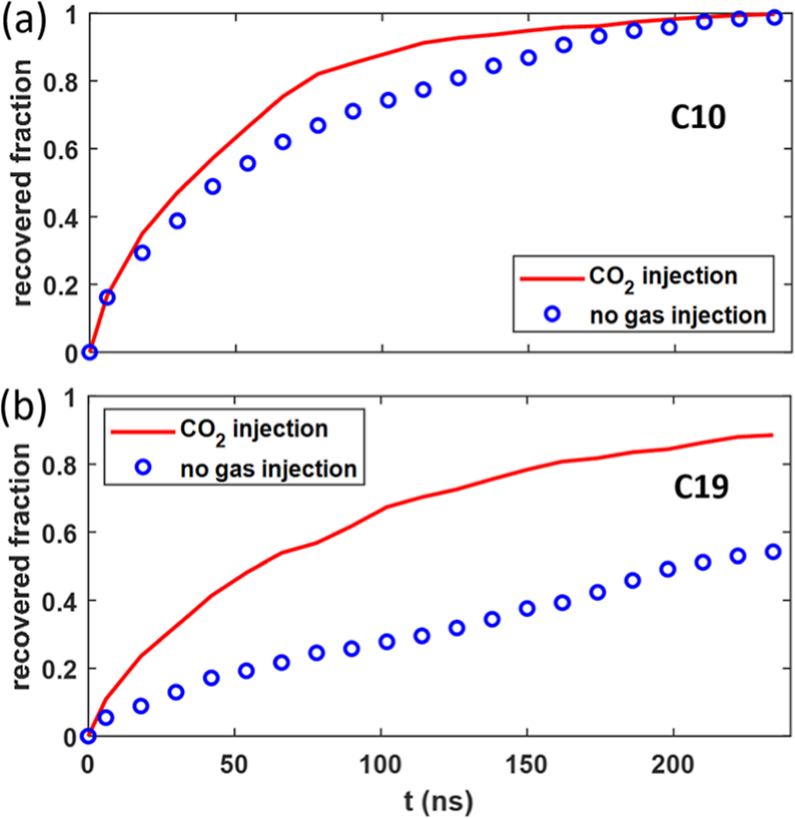
(a,b) Evolution of the fraction of C10 molecules (a) and C19 molecules (b) recovered during the oil recovery simulations performed with and without CO_2_ injection.

Figure 5 shows the evolution of the fractions of C10 and C19 recovered from the pore when the gas bath is filled with CO_2_. Oil recovery is greatly accelerated by CO_2_ injection: compared to the case in Section 3.1, for C10, the time for a 90% recovery has decreased from 161 to 107 ns; for C19, a 55.1% recovery is achieved at 73 ns instead of 240 ns. Further, the undesired selectivity of recovery is mitigated by CO_2_ injection: as shown in Figure 3c, the selectivity factor has been lowered to 1.5 initially and to 1.1 as time approaches 240 ns.

**Figure 6:**
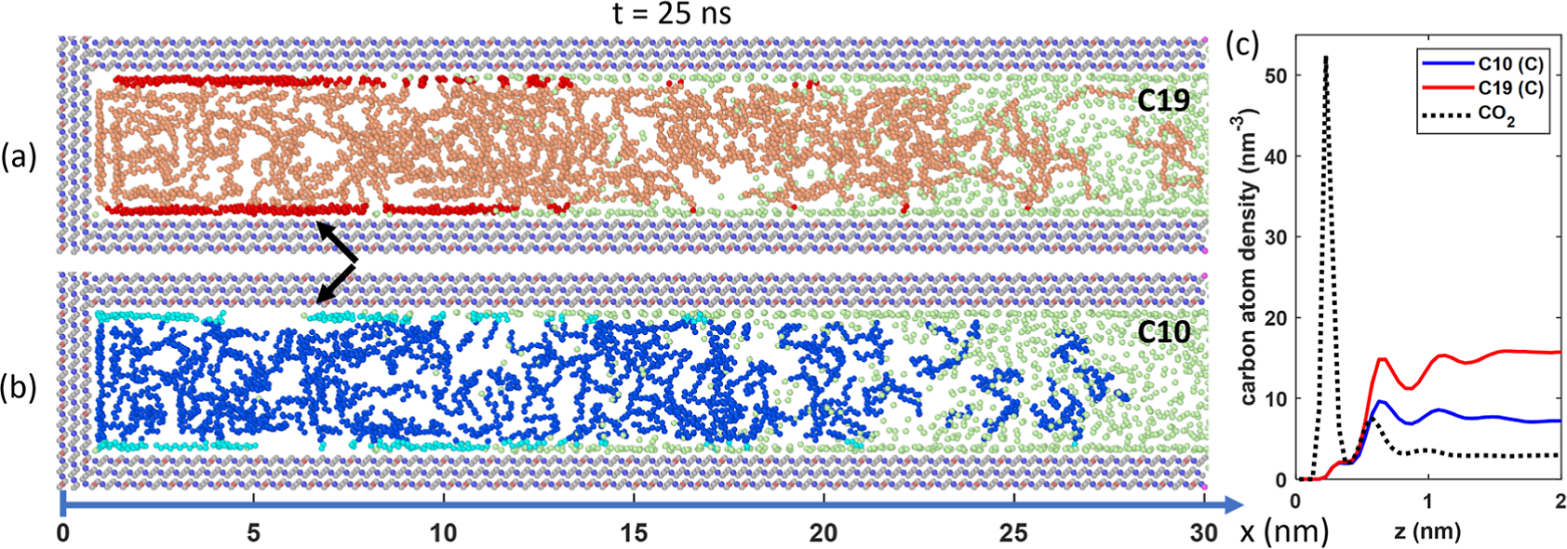
(a,b) Side-view snapshots of the calcite pore and fluids inside it at *t* = 25 ns of the oil recovery simulation when the gas bath is filled with CO_2_ at 345 bar. In (a), the red and light red denote the adsorbed and free C19 molecules. In (b), light blue and blue denote the adsorbed and free C10 molecules. The C atoms of the CO_2_ molecules are shown as green dots. The black arrow indicates the approximate location of the CO_2_ diffusion front. (c) Density profiles of C10, C19, and CO_2_ across the calcite pore averaged in the space behind the CO_2_ diffusion front (7 nm < *x* < 30 nm) at *t* = 25 ns.

We can qualitatively understand the acceleration of oil recovery and mitigation of recovery selectivity by examining the molecular processes underlying these phenomena. Visualization of the trajectories reveals that, at *t* > 0, CO_2_ molecules move toward the pore’s interior in a diffusion-like mode (see Figure S2 in the Supporting Information). The diffusion-like transport of CO_2_ is similar to that reported in recent MD works.^24,25,47,48^ A representative snapshot of the system taken at *t* = 25 ns is shown in Figure 6. A CO_2_ diffusion front is observed at *x* ≈ 7 nm. Near this diffusion front, where the CO_2_ loading is low, CO_2_ is mainly absorbed on the pore walls. As we move from the diffusion front toward the pore opening, the CO_2_ loading increases, and CO_2_ molecules are observed across the entire pore. CO_2_ can displace C10 and C19 molecules from the pore walls, especially in the region far behind the diffusion front. This is seen in Figure 6c, where the average C10, C19, and CO_2_ density across the pore behind the diffusion front (i.e., in the region 7 nm < *x* < 30 nm) at *t* = 25 ns is shown. The more competitive adsorption of CO_2_ on calcite pore walls is well-known. It can be attributed to the electrostatic quadrupole-charge interactions between CO_2_ molecules and the ionic sites on calcite surfaces.^24,25,27,47,49^ The density peaks of CO_2_ molecules are slightly closer to the wall than oil due to their smaller size along the minor axis compared to the alkane molecules.

C10 and C19 molecules displaced from the pore walls enter the bulk portion of the pore, where CO_2_ is mixed well with C10 and C19 molecules to serve as a solvent for them. This solubilization effect allows the oil molecules across the entire pore width to transport toward the pore opening to be recovered rather thoroughly than through the adsorption layer on pore walls, as in Section 3.1. Consequently, oil recovery is accelerated. The bulk solubilization and elimination of adsorbed oils are more beneficial for C19 recovery because the recovery of C19 molecules is hindered more by surface adsorption in the no-gas-injection scenario in Section 3.1. Consequently, the preferential recovery of C10 over C19 is mitigated, as shown in Figure 3c.

We can gain more insights into the CO_2_ EOR by examining the spatiotemporal evolution of CO_2_, C10, and C19 in the pore and how the amount of adsorbed and free oil is reduced. Because CO_2_ molecules adsorb strongly on pore walls (see Figure 6c), we also divide them into the adsorbed (less than 0.38 nm from the pore wall) and free (more than 0.38 nm from the pore wall) populations.

**Figure 7:**
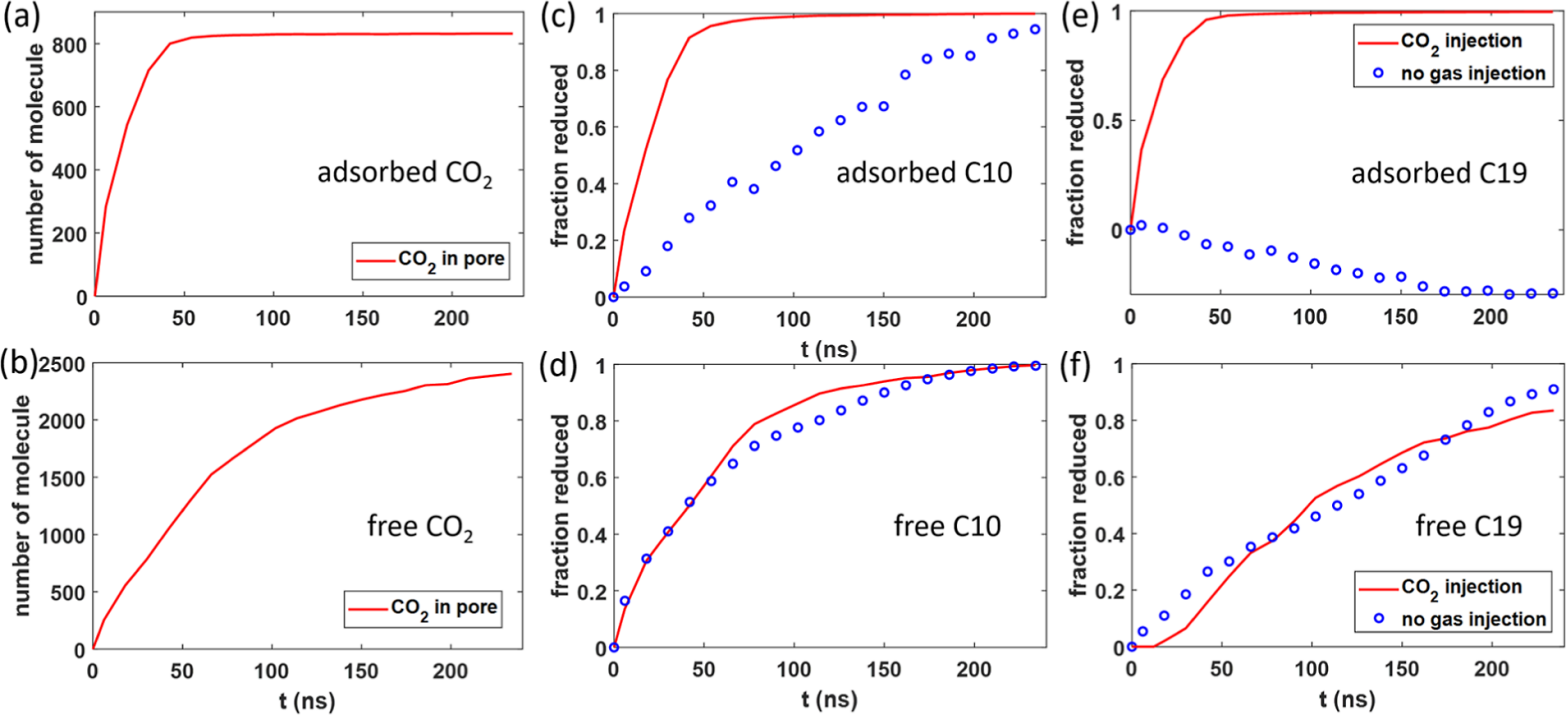
Oil recovery in the presence of CO2injection. (a,b) Evolution of the number of adsorbed (a) and free (b) CO_2_ molecules inside the calcite pore. (c,d) Evolution of the reduction of the fraction of adsorbed (c) and free (d) C10 molecules inside the calcite pore during oil recovery. (e,f) Evolution of the reduction of the fraction of adsorbed (e) and free (f) C19 molecules inside the calcite pore during oil recovery.

**Figure 8:**
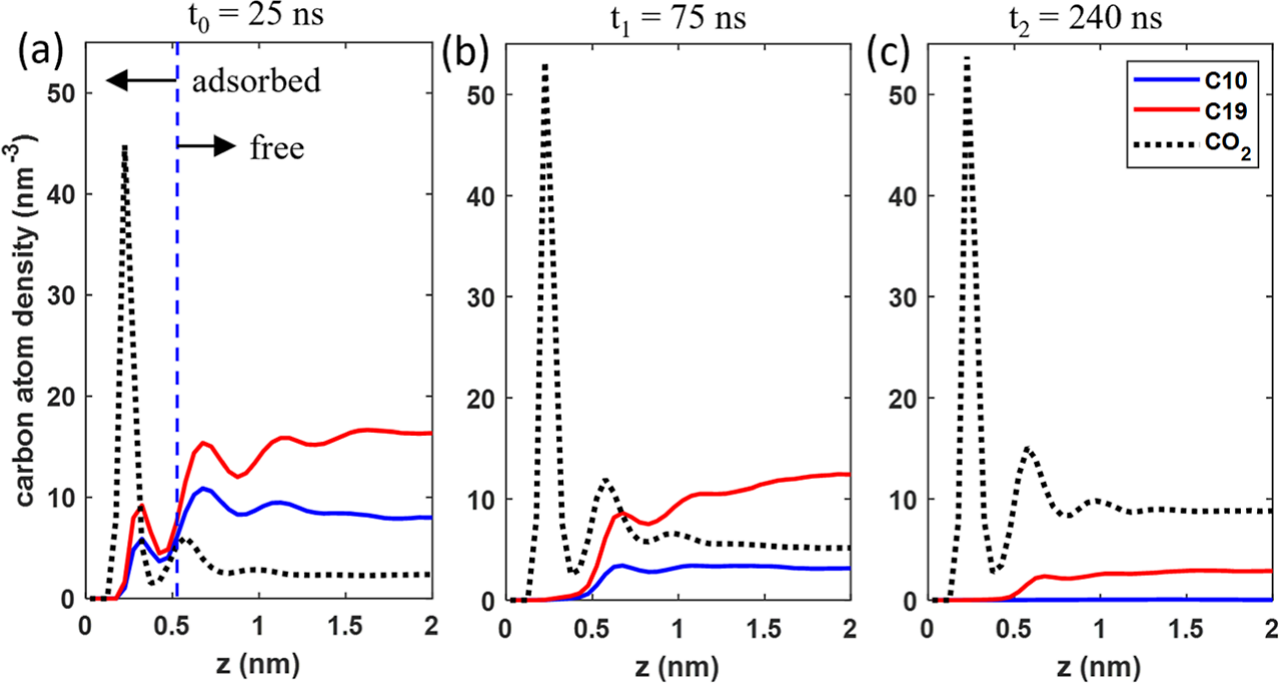
(a,c) Density profiles of C10, C19, and CO_2_ across the calcite pore averaged inside the entire pore at *t* = 25 (a), 75 (b), and 240 ns (c).

During the early stage of oil recovery (*t* < 25 ns), the CO_2_ diffusion front moves rapidly toward the pore’s dead end. A significant fraction of the CO_2_ in the pore becomes adsorbed on pore walls due to its stronger affinity to pore walls than to bulk oil. The adsorbed CO_2_ thus approaches its final state more rapidly than the free CO_2_ (cf. Figure 7a,b). By *t* = 25 ns, the CO_2_ diffusion front has reached ∼7 nm from the pore’s end (see Figure 6a), and the adsorbed CO_2_ has reached 78.6% of its saturation value. The adsorbed CO_2_ displaces adsorbed C10 and C19 off pore walls, which tends to elevate C10 and C19 densities in the bulk portion. Indeed, a comparison of Figures 2a and 8a shows that, despite some C19 molecules being recovered from the pore by *t* = 25 ns, the average C19 density in the pore’s center at this time is *higher* than its initial value. While the CO_2_ entering the pore solubilizes the oil and facilitates their recovery, as discussed above, Figure 5a shows that the recovery factor of C10 only increases from 34.5% in the no-gas-injection case to 42.4% by *t* = 25 ns. A more detailed look at the recovery of different oil populations (see Figure 7c,d) reveals that, with CO_2_ injection, by *t* = 25 ns, 63.7% of adsorbed C10 has been removed from pore walls (compared to 9.2% in the no-gas-injection case), but the reduction of the free C10 inside the pore is nearly the same as in the no-gas-injection case.

To understand the marginal improvement of C10 recovery due to CO_2_ injection at *t* < 25 ns, recall that the flux of C10 inside a pore containing C10, C19, and CO_2_ (denoted as species 1, 2, and 3) can be given by^29,50−52^*J*_1_ = −Γ_11_∇μ_1_ – Γ_12_∇μ_2_ – Γ_13_∇μ_3_ according to nonequilibrium thermodynamics (here, the Onsager approach is preferred over the classical Fickian approach since it allows the coupling between the transport of different components to be more easily represented).^53,54^ Here, μ_*i*_ is the chemical potential of species *i*. Γ_*ij*_ is the Onsager coefficient for the species pair (*i,j*), which depends on the concentration of these species and their interactions. As can be expected from the limiting case of self-diffusion, Γ_*ii*_ tends to increase as the mobility of species *i* increases.^51^

In the no-gas-injection case, C10 recovery is caused by the transport of the adsorbed C10 in the liquid films behind the meniscus, and thus, the density of C10 transporting toward the pore opening is low. CO_2_ allows C10 to be solubilized in the pore’s bulk portion, which leads to an increased density of C10 transporting toward the gas bath. For example, at *t* = 25 ns, the cross-section-averaged C10 density in the space behind the CO_2_ diffusion front is 0.60 nm^–3^, which is enhanced 3.53 times over the cross-section-averaged density of 0.17 nm^–3^ contributed by C10 in the liquid film behind the meniscus shown in Figure 4c (if only C10 in the more mobile, second adsorption layer of the liquid films in Figure 4c is considered, the enhancement factor is 6.0). Such an enhancement of C10 concentration helps increase Γ_11_ and thus C10 transport out of the pore. However, two other factors tend to reduce the C10 recovery. First, during the early stage of C10 recovery, the pore is characterized by a dispersion of CO_2_ molecules in dense liquid oils. The interactions between C10 molecules and their surrounding C19 and CO_2_ molecules tend to reduce C10s mobility, which tends to reduce Γ_11_ and limit C10 recovery. Second, the ingression of CO_2_ into the pore tends to drive C10 molecules toward the pore’s dead end, thus suppressing their recovery. Given these two competing factors, the enhancement of C10 recovery by CO_2_ injection becomes limited.

In sharp contrast to C10, the recovery of C19 during the early stage of oil recovery is enhanced significantly by the CO_2_ injection. Figure 5b shows that at *t* = 25 ns, the recovery of C19 has increased from 11.5 to 28.1% due to CO_2_ injection. Figure 7e further reveals that 82.3% of adsorbed C19 has been removed from the pore walls. Because a large portion of the displaced C19 molecules remains inside the pore as free C19, the total free C19 only reduces by 3.3% from its initial value (Figure 7f).

The accelerated recovery of C19 mainly originates from the significant increase in the number of highly mobile C19 molecules that can be transported out of the pore. For example, at *t* = 25 ns, the displacement of C19 molecules from pore walls to the pore’s bulk portion leads to a cross-section-averaged C19 density of 0.58 nm^–3^ behind the CO_2_ diffusion front. This density is almost 10 times the cross-section-averaged density of 0.06 nm^–3^ contributed by the second C19 adsorption layer in the liquid film behind the meniscus shown in Figure 4c. Similar to C10 recovery, C19 recovery is also hampered by the dense molecular packing in the oil–CO_2_ mixture and the movement of CO_2_ toward the pore’s dead end. However, these factors appear far less important compared to the strong enhancement of C19 participating in the recovery process. Overall, the greater acceleration of C19 recovery than C10 at *t* < 25 ns reduces the selectivity factor from 3.0 in the no-gas-injection case to 1.5 when CO_2_ is injected, as seen in Figure 3c.

At *t* ∼ 30 ns, the CO_2_ diffusion front reaches the pore’s dead end, and most of the adsorbed C10 and C19 molecules are removed from the pore walls by *t* = 75 ns. The average density profiles in Figure 8b show that the C10 and C19 molecules are now separated from the pore walls by a dense CO_2_ layer. From *t* = 25 to 75 ns, the recovery of C10 increases from 42.4 to 80.8%, considerably higher than when there is no gas injection. This better enhancement of C10 recovery than in the initial stage (*t* < 25 ns) can be attributed to the enhancement of C10 mobility as time increases. As shown in Figure 8b, at *t* = 75 ns, the CO_2_ density inside the pore has exceeded that of C10 and reached about half of C19. Such a CO_2_–oil mixture is much less viscous than that at *t* = 25 ns. Therefore, the mobility and thus the recovery of C10 molecules increase markedly. The recovery of C19 likewise shows a great enhancement over the no-gas-injection case from *t* = 25 to 75 ns for a similar reason. Over this period, the selectivity factor was reduced only marginally.

As the recovery process reaches *t* = 240 ns, all C10 molecules are removed, and only about 18% of free C19 remains inside the pore (see Figure 7c–f). The latter leads to an overall recovery of 89% for C19, compared to 55% when there is no gas injection (Figure 5). At *t* = 240 ns, the oil and CO_2_ density profiles shown in Figure 8c suggest that the pore essentially contains a dilute solution of C19 molecules in gaseous CO_2_. The entropic gain driving the transport of C19 from the pore to the gas bath thus becomes small, and further recovery of C19 is sluggish.

All of the above results are obtained for calcite nanopores of 4 nm width. The selective recovery of the C10 and C19 mixtures will differ for different pore widths. As the pore width decreases, the interfacial effects become stronger. For example, a larger portion of C19 in the pore will be adsorbed on the pore walls, hindering their recovery. So, the selectivity of C10 over C19 likely will be enhanced in the absence of gas injection. However, because a more significant fraction of injected CO_2_ will adsorb on pore walls to displace adsorbed C19, the mitigation of C10 selectivity should be more significant as pore width decreases. The opposite trend will likely occur as the pore width increases. Studies on how pore size affects recovery selectivity should be pursued in the future to obtain a thorough understanding of the pore width effects.

## 4 Conclusions

In summary, using molecular dynamics simulations, the recovery of an oil mixture composed of C10 and C19 from a single 4 nm-wide calcite pore is studied with and without CO_2_ injection. A large fraction of oil molecules is initially stored in the pore as adsorbed molecules, especially for the heavier C19. In the absence of gas injection, oil is recovered at rates much higher than expected from the oil vaporization mechanism alone, and a strong preferential recovery of C10 is observed. Oil recovery occurs through the diffusion of oil from the liquid meniscus along the molecularly thin liquid films on pore walls to the bath. The strong oil–wall interactions lead to a high oil density on walls to facilitate recovery but also reduce the mobility of oil molecules directly adsorbed on pore walls to hinder oil recovery. The latter is especially important for the heavier C19, which manifests as a stronger selectivity toward the lighter C10 during recovery.

When CO_2_ is injected under miscible conditions, oil recovery is significantly accelerated and the recovery selectivity toward C10 is mitigated. CO_2_ molecules entering the pore rapidly displace oil molecules adsorbed on pore walls, and the CO_2_ remaining in the pore’s bulk portion solubilizes C10 and C19. The latter dramatically increases the number of mobile oil molecules participating in recovery compared with the no-gas-injection case, where oil transport is largely a surface phenomenon. The CO_2_ solubilization mechanism thus contributes decisively to the EOR. Compared to C10, a larger fraction of C19 is adsorbed on pore walls initially and has a lower mobility. Therefore, the enhancement of C19 recovery by the solubilization mechanism is more distinct than that of C10, leading to a reduced selectivity of C10 recovery compared to the no-gas-injection case.

The results from our simulations highlight the crucial role of interfacial phenomena (in particular, competitive adsorption of oil and gas molecules and modulation of molecular transport properties by adsorption) in the recovery of oil from nanopores. Fundamentally, these phenomena and their relative significance compared to bulk phenomena depend on a host of factors not explored here, e.g., pore width, chemical nature of pore walls (e.g., whether walls are made of kerogen or minerals such as quartz and mica), existence of connate water, and type of gas injected (e.g., CO_2_, CH_4_, N_2_, ...). For example, at low connate water saturation, water exists as liquid films on the pore walls. These water films, being more polar than oil and CO_2_, displace oil from the pore walls but cannot be displaced by injected CO_2_. Therefore, the enhanced recovery of C19 over C10 due to the displacement of adsorbed C19 by CO_2_ molecules will disappear, and the efficacy of the CO_2_ injection in mitigating the preferential recovery of C10 over C19 may decrease. Further pore-scale MD simulations are warranted to explore how these factors affect the interfacial phenomena revealed here.

Ultimately, for pore-scale MD studies to benefit gas injection-based EOR at the reserve scale, it is helpful to combine MD studies with analytical and numerical models at larger scales (e.g., lattice Boltzmann or pore network models). Here, MD results can guide the development of analytical models, furnish the thermodynamic and transport properties needed by those models, and serve as benchmarks for testing their performance. It is encouraging that these aspects have already received attention in the literature.^55,56^

## Supporting Information Available

Full snapshot of the simulation system; side-view snapshots of the fluids inside the calcite pore at different times with CO_2_ in the gas bath; and force field parameters for all molecules (PDF)
