# Supplementary material for: Enhanced Recovery of Oil Mixtures from Calcite Nanopores Facilitated by CO2 Injection
Source: Energy Fuels. 2024 Mar 8;38(6):5172–82. doi: 10.1021/acs.energyfuels.3c05235 (PMC10961724; doi:10.1021/acs.energyfuels.3c05235)
Supplement: Supplementary file 1 — ef3c05235_si_001.pdf [file ef3c05235_si_001.pdf]

Supporting Information

Enhanced Recovery of Oil Mixtures from Calcite Nanopores Facilitated by CO_2 Injection

Hongwei Zhang,[1] Shihao Wang,[2] Xin Wang,[1] and Rui Qiao[1,*]

[1] Department of Mechanical Engineering, Virginia Tech, Blacksburg, VA 24061, United States
[2] Chevron Technical Center, Chevron, Houston TX, 77002, United States

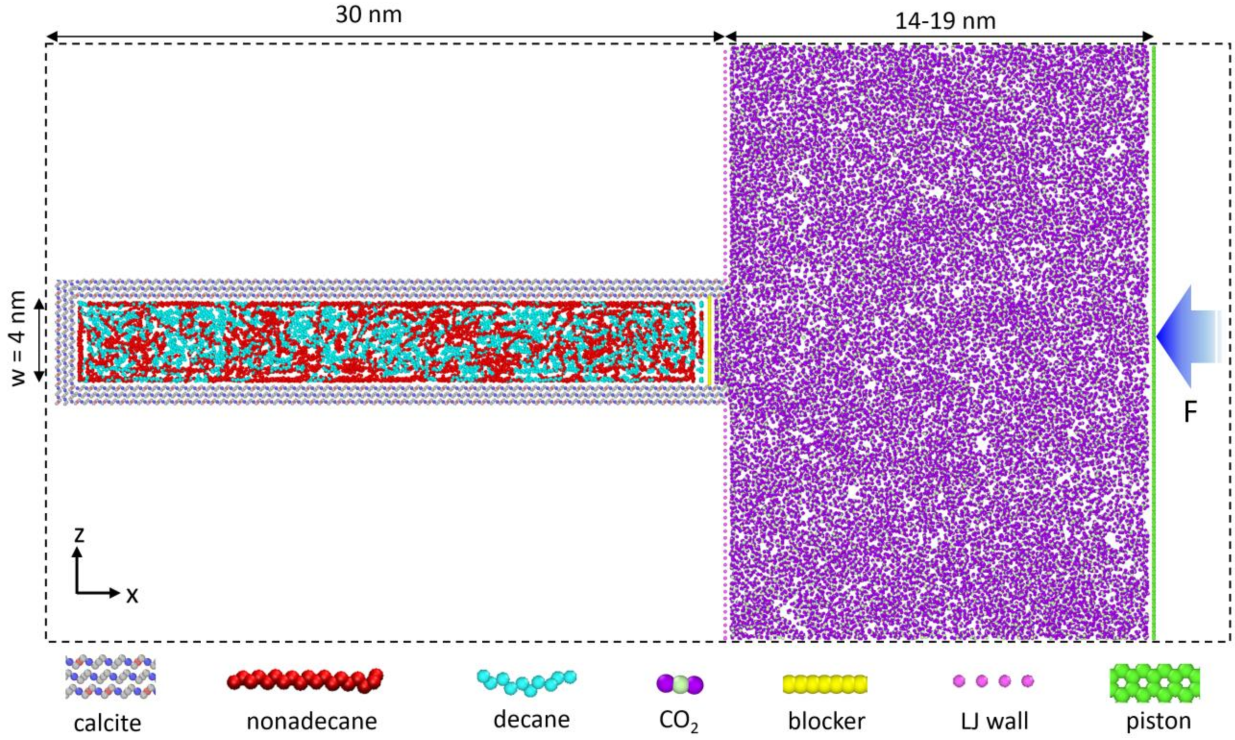

Figure S1. A snapshot of the simulation system for studying the recovery of decane+nonadecane (C10+C19) mixtures from a single calcite nanopore aided by CO_2 injection. The system measures 29.46 nm in the z-direction. The dashed black lines denote the simulation box.

* To whom correspondence should be addressed. Email: ruiqiao@vt.edu

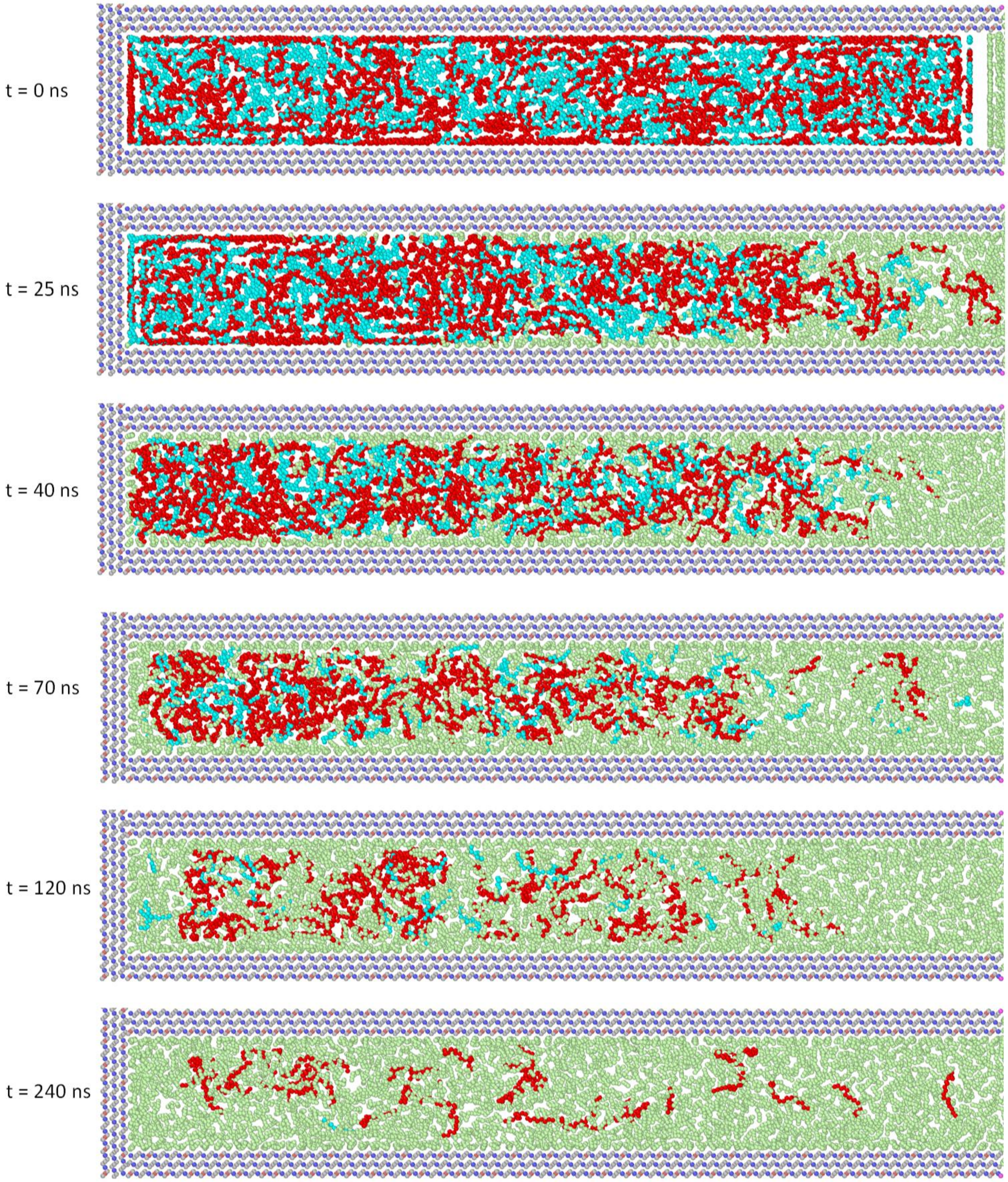

Figure S2. Side-view snapshots of the calcite pore and fluids inside it at different times of the oil recovery simulation when the gas bath is filled with CO_2 at 345 bar. The red and blue denote C19 and C10 molecules, respectively. The C atoms of CO_2 molecules are shown as green dots.

Table S1. Force field parameters

Atom Type	Mass (g/mol)	σ (Å)	ε (kcal/mol)	Charge (e)
C ($CaCO_3$)	12.011	3.39967	0.08600	1.04085
Ca ($CaCO_3$)	40.078	3.05240	0.04598	1.64203
O ($CaCO_3$)	15.999	2.95992	0.21000	-0.89429
C (CO_2)	12.011	2.800	0.055927	0.65160
O (CO_2)	15.999	3.028	0.159704	-0.32580
CH_2 (C10/C19)	14.027	3.930	0.09101338	0
CH_3 (C10/C19)	15.035	3.910	0.20666826	0
Piston	12.011	3.31500	0.00576	0
Blocker	12.011	3.27000	0.25466	0

Molecule Type	bond_coeff K (kcal/mol Å^2)/ r_0 (Å^2)	angle_coeff K (kcal/mol) / θ_0 (°)	dihedral_coeff (kcal/mol)
CO_2	71.7017 / 1.162	13.1453 / 180.0	-
C10/C19	95.8825 / 1.54	62.0997 / 114.0	2.0070 -4.012 0.2710 6.2901

The interaction between CO_2 and alkane are calculated by the modified Lorentz-Berthelot rule:

$$\sigma_{ij} = \frac{\sigma_{ii} + \sigma_{jj}}{2}; \ \ \varepsilon_{ij} = a\sqrt{\varepsilon_{ii}\varepsilon_{jj}}$$

with a = 0.9 here.
